# Mechanical dysfunction: ovarian cancer ascites disables NK cells by suppressing CDC42-driven force generation

**DOI:** 10.3389/fimmu.2026.1883517

**Published:** 2026-10-16

**Authors:** Chenyi An, Yujun Xu, Ziyu Zhou, Jiong Wang, Xuan Liu, Ting Guo, Zhihua Yan, Lvyang Zhou, Xing Zhao, Rui Qin, Zhu Zeng, Shi Zuo

**Affiliations:** 1 Department of Hepatobiliary Surgery, Key Laboratory for Cancer Prevention and Treatment of Guizhou Province, The Affiliated Hospital of Guizhou Medical University, School of Biology and Engineering (School of Modern Industry for Health and Medicine), Guizhou Medical University, Guiyang, China; 2 Department of Immunology, School of Basic Medical Sciences, Guizhou Medical University, Guiyang, China; 3 Obstetrics and Gynecology Department, The Affiliated Hospital of Guizhou Medical University, Guiyang, China; 4 Tissue Engineering and Stem Cell Experiment Center, Guizhou Medical University, Guiyang, China; 5 Guizhou Medical Sequencing Center, Precision Medicine Research Institute of Guizhou, The Affiliated Hospital of Guizhou Medical University, Guiyang, China; 6 Key Laboratory of Infectious Immune and Antibody Engineering of Guizhou Province, Engineering Research Center of Cellular Immunotherapy of Guizhou Province, Guizhou Key Laboratory of Microbio and Infectious Disease Prevention & Control, Guizhou Medical University, Guiyang, China

**Keywords:** CDC42, malignant ascites, mechanobiology, natural killer cells, ovarian cancer, traction force

## Abstract

**Introduction:**

Ovarian cancer ascites creates an immunosuppressive microenvironment that compromises NK cell function, yet the underlying mechanisms remain incompletely understood.

**Methods:**

We used ascites-conditioned NK cells and assessed their mechanophenotypes, including F-actin, cortical tension, and traction force, along with CDC42 activity. Transcriptomic analysis and bidirectional pharmacological manipulation of CDC42 were performed.

**Results:**

Ascites-conditioned NK cells exhibited a paradoxical cytoskeletal state—elevated F-actin yet reduced cortical tension and traction force—indicating a failure to translate actin polymerization into productive mechanical output. Mechanistically, ascites triggered de novo expression of the CDC42 negative regulators, CDC42EP1 and CDC42EP5, leading to suppressed CDC42 activity. Bidirectional pharmacological manipulation of CDC42 reciprocally regulated traction force and cytotoxicity, establishing a causal link between CDC42-dependent mechanics and NK cell killing. The near-perfect correlation between traction force and killing capacity positions traction force as a robust mechanobiomarker of NK cell competence.

**Discussion:**

These findings uncover a previously unrecognized role for CDC42 in governing NK cell mechanics and identify CDC42 as a therapeutically actionable node for restoring anti-tumor immunity in immunosuppressive microenvironments.

## Introduction

Ovarian cancer remains the most lethal gynecological malignancy worldwide, with a five-year survival rate of approximately 30% for advanced-stage disease (1). This poor prognosis is largely attributable to late-stage diagnosis and the frequent development of resistance to platinum-based chemotherapy (1). A hallmark of advanced ovarian cancer is the accumulation of malignant ascites within the peritoneal cavity, a protein-rich fluid that creates a unique and dynamic tumor microenvironment (1–3). Ascites is not merely a passive bystander but an active ecosystem that enforces vascular leakage, fuels cellular reprogramming, and establishes profound immune suppression, thereby driving peritoneal dissemination, therapy resistance, and poor clinical outcomes (1). Single-cell profiling has recently revealed the complex cellular landscape of ascites, comprising functionally distinct malignant, stromal, and immune populations that interact to shape disease progression (4). Despite advances in immunotherapy, clinical responses in ovarian cancer remain limited, underscoring the urgent need to better understand how the ascites microenvironment subverts anti-tumor immunity (2).

Natural killer (NK) cells are innate lymphocytes capable of recognizing and eliminating tumor cells without prior sensitization, positioning them as promising effectors for cancer immunotherapy (5). However, NK cells isolated from ovarian cancer ascites exhibit profound functional impairments, including reduced cytokine production, decreased cytotoxic granule release, and diminished killing capacity (6, 7). Recent studies have identified multiple mechanisms contributing to this dysfunction: ascites-derived factors suppress NK cell metabolism and effector function (2); uptake of polar lipids, particularly phosphatidylcholine, disrupts NK cell membrane order and cytotoxicity (2, 8); and high protein concentrations and immunoglobulins in ascites impair NK cell degranulation and target cell conjugation (9). While these biochemical signals clearly contribute to NK cell suppression, emerging evidence suggests that the tumor microenvironment may also compromise NK cell function by altering their physical and structural properties (10, 11). For instance, a recent study by Zheng et al. demonstrated that intratumoral NK cells from human liver cancer exhibit a striking loss of membrane protrusions and reduced F‐actin content, changes that correlate with impaired immune synapse formation and cytotoxicity (11). Although the observed membrane alterations were attributed to dysregulated serine metabolism leading to sphingomyelin depletion (11), the concomitant reduction in F‐actin points to a potential biomechanical dimension of NK cell dysfunction within tumors.

The emerging field of mechanoimmunology has established that immune cell effector functions are not solely governed by biochemical signals but are also critically dependent on physical and mechanical processes (12–14). A series of landmark studies have revealed that cytotoxic lymphocytes engage in mechanosurveillance, a process whereby immune cells sense and respond to the biophysical characteristics of cancer cells to eliminate them (15–17). Early work from the Huse and Er groups demonstrated that cancer cells with increased stiffness, driven by MRTF-mediated actin cytoskeleton remodeling, trigger stronger cytotoxic T lymphocyte and NK cell activation, establishing that target cell mechanics serve as a critical determinant of immune vulnerability (15). This paradigm has been further extended by recent groundbreaking work from Ni, Jiang and colleagues, who demonstrated that leukemia stem cells evade NK cell immunosurveillance through a “soft and small” biomechanical phenotype, and that pharmacologically induced stiffening of these target cells enhances immune synapse formation and cytotoxic efficacy (18). While these studies have illuminated how the mechanical properties of target cells influence immune recognition, an equally important question is whether the intrinsic mechanical competence of immune cells themselves is similarly regulated and can be dysregulated in pathological settings. This perspective has given rise to the concept of mechano-immunological checkpoints, wherein the mechanophenotypes of immune cells, such as cytoskeletal organization, cortical tension, and traction force generation, are intimately coupled with their immunophenotypes and represent previously unexplored nodes for therapeutic intervention (13).

For NK cells, dynamic reorganization of the actin cytoskeleton and the generation of cytoskeletal forces are essential for multiple effector functions, including target cell recognition, immune synapse maturation, and cytotoxic granule delivery (12, 19, 20). These biomechanical events are tightly regulated by Rho family GTPases, particularly CDC42, a master organizer of cytoskeletal dynamics, cell polarity, and actin polymerization (20–22). In cytotoxic lymphocytes, CDC42 governs the transience of polarization, a process characterized by the rapid recruitment of actin to the immune synapse followed by its timely release (20). This spatiotemporal precision is critical for preventing nonproductive target cell interactions and enabling sequential killing. Moreover, dysregulation of CDC42 activity has been linked to impaired NK cell function in the context of aging (23). However, whether and how the ovarian cancer ascites microenvironment perturbs NK cell function through mechanical aspects, particularly the CDC42-mediated force generation, remains largely unexplored. In this study, we uncover a previously unrecognized mechano-immunological mechanism by which ovarian cancer ascites suppresses NK cell cytotoxicity. We demonstrate that ascites exposure induces a paradoxical cytoskeletal state in NK cells, characterized by elevated F-actin content yet reduced cortical tension and traction force, indicating a failure to translate actin polymerization into productive mechanical output. Mechanistically, this dysfunction is driven by the *de novo* induction of the CDC42 negative regulators CDC42EP1 and CDC42EP5, leading to suppressed CDC42 activity. Bidirectional pharmacological manipulation of CDC42 establishes a causal link between CDC42-dependent mechanics and NK cell killing capacity, positioning traction force as a robust mechanobiomarker and CDC42 as a therapeutically actionable node. Our findings introduce a mechano-immunological framework for understanding NK cell suppression in the tumor microenvironment and open new avenues for restoring anti-tumor immunity through targeting immune cell mechanics.

## Results

### Ovarian cancer ascites impairs NK cell cytotoxicity

We firstly pretreated NK-92MI cells (denoted as NK cell for short hereafter) with ascites from ovarian cancer patients for 24 hours to generate ascites-conditioned NK cells (AS-NK), and then cocultured them with SKOV3 cells at effector-to-target (E:T) ratios ranging from 2:1 to 16:1 for 5 hours, after which NK cell cytotoxicity and effector molecule expression were assessed (**Figure 1A**). Ascites treatment significantly attenuated NK cell cytotoxicity across different E:T ratios, where the percentages of apoptotic SKOV3 cells (Annexin V^+^/PI^-^ and Annexin V^+^/PI^+^) exhibited a clear reduction compared to those of untreated NK cells (NK), demonstrating a profound suppression of NK cell cytotoxicity in ovarian cancer ascites (**Figures 1B, C**, **Supplementary Figure 1A**). To further investigate the mechanisms underlying NK cell dysfunction induced by ovarian cancer ascites, we compared key effector alterations between AS-NK and untreated NK cells. Consistent with the impaired killing, AS-NK cells showed significantly reduced IFN-γ secretion (**Figure 1D**) and a lower percentage of perforin-positive cells (**Figure 1E**), whereas GZMB expression remained unchanged (**Supplementary Figures 1B, C**). In addition, we found no significant differences in CD107a expression (**Supplementary Figure 1D**) or extracellular concentrations of secreted perforin and GZMB (**Supplementary Figures 1E, F**) between the two groups. Together, these results indicate that ascites pretreatment selectively suppresses IFN-γ and intracellular perforin expression without broadly affecting other cytotoxic granule-associated readouts, suggesting a preferential impairment of specific effector components rather than a global shutdown of the cytotoxic machinery.

**Figure 1:**
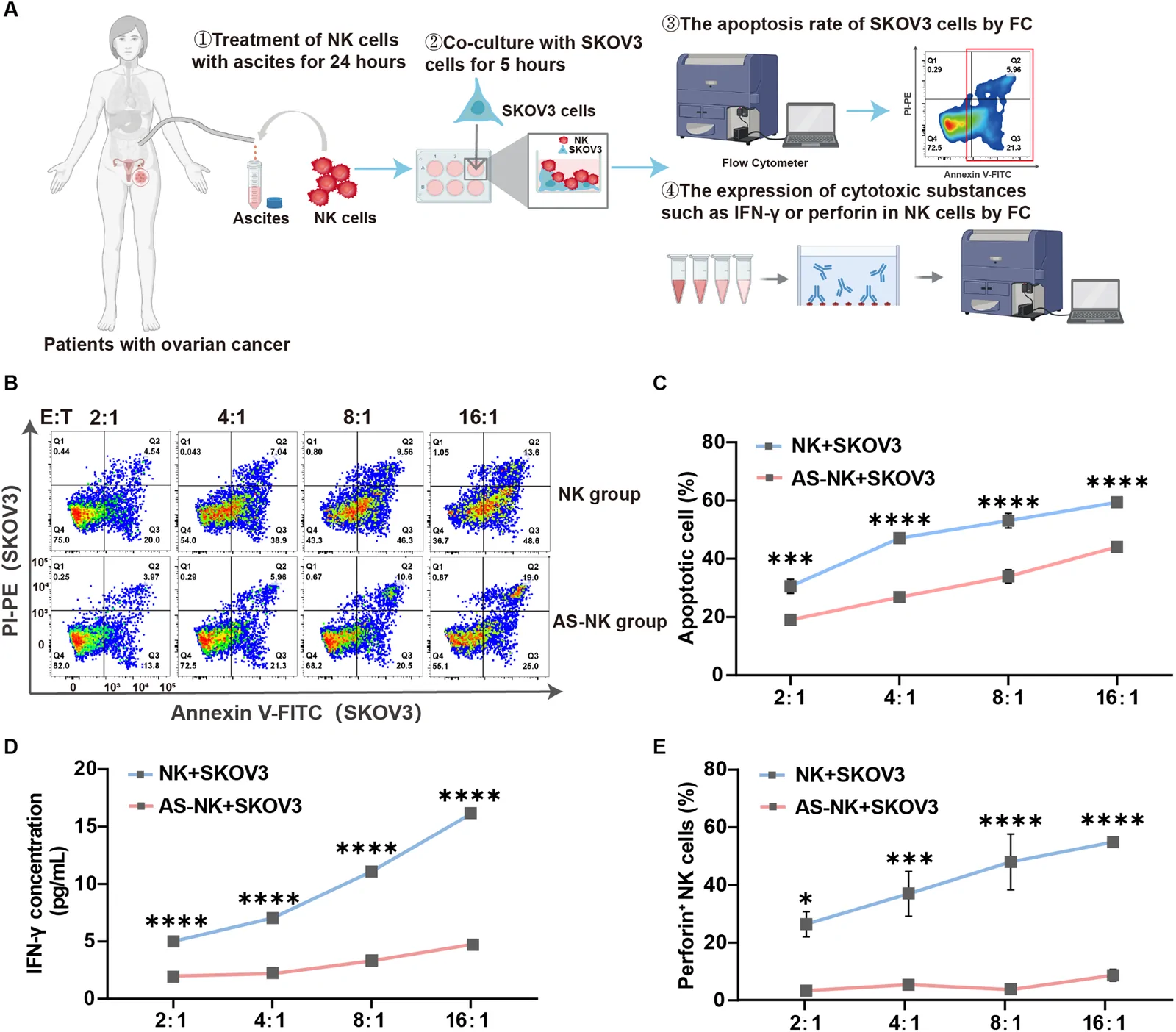
Ascites pretreatment impairs NK cell cytotoxicity. **(A)** Schematic diagram for evaluating the effects of ovarian cancer ascites on NK-92MI cell cytotoxicity against SKOV3 cells. **(B, C)** Representative flow cytometry plots **(B)** and quantification **(C)** of apoptotic SKOV3 cells (Q2+Q3) at the indicated effector-to-target (E:T) ratios. **(D, E)** Quantification of IFN-γ concentration in culture supernatants **(D)** and the percentage of perforin-positive NK cells **(E)** after co-culture under the same conditions. *, *** and **** refer to *p* < 0.05*, p* < 0.001 and *p* < 0.0001, respectively in Two-way ANOVA analysis, data are presented as mean ± s.e.m. of *n* = 3 independent experiments.

### Ovarian cancer ascites remodels the mechanophenotypes of NK cells

Given that efficient NK-cell killing requires coordinated cytoskeletal remodeling and force generation (12, 19, 20, 24), we next asked whether the loss of cytotoxicity induced by ascites is accompanied by alterations in NK-cell mechanophenotypes. Scanning electron microscopy (SEM) revealed that NK cells conditioned with ovarian cancer ascites for 24 hours adopted a significantly more rounded morphology with fewer and blunted membrane protrusions (**Figures 2A–C**). This observation aligns with prior reports that NK cells residing within the tumor microenvironment (TME) of various solid malignancies, including hepatocellular, lung, colon, and ovarian carcinomas, exhibit a similarly smoothed and rounded morphology (11). However, our findings diverged sharply from those in solid tumors: while F-actin expression was reduced in solid tumor-infiltrating NK cells (11), it was elevated in ascites-conditioned NK cells (**Figures 2D, E**). Consistently, AFM measurements, which quantify cellular stiffness by probing the force-distance relationship between the AFM tip and the cell surface, showed that ascites-conditioned NK cells exhibited a significant increase in stiffness (**Figures 2F, G**). This dichotomy suggests that the liquid ascites microenvironment and the solid TME may employ distinct biomechanical strategies to suppress NK cell function.

**Figure 2:**
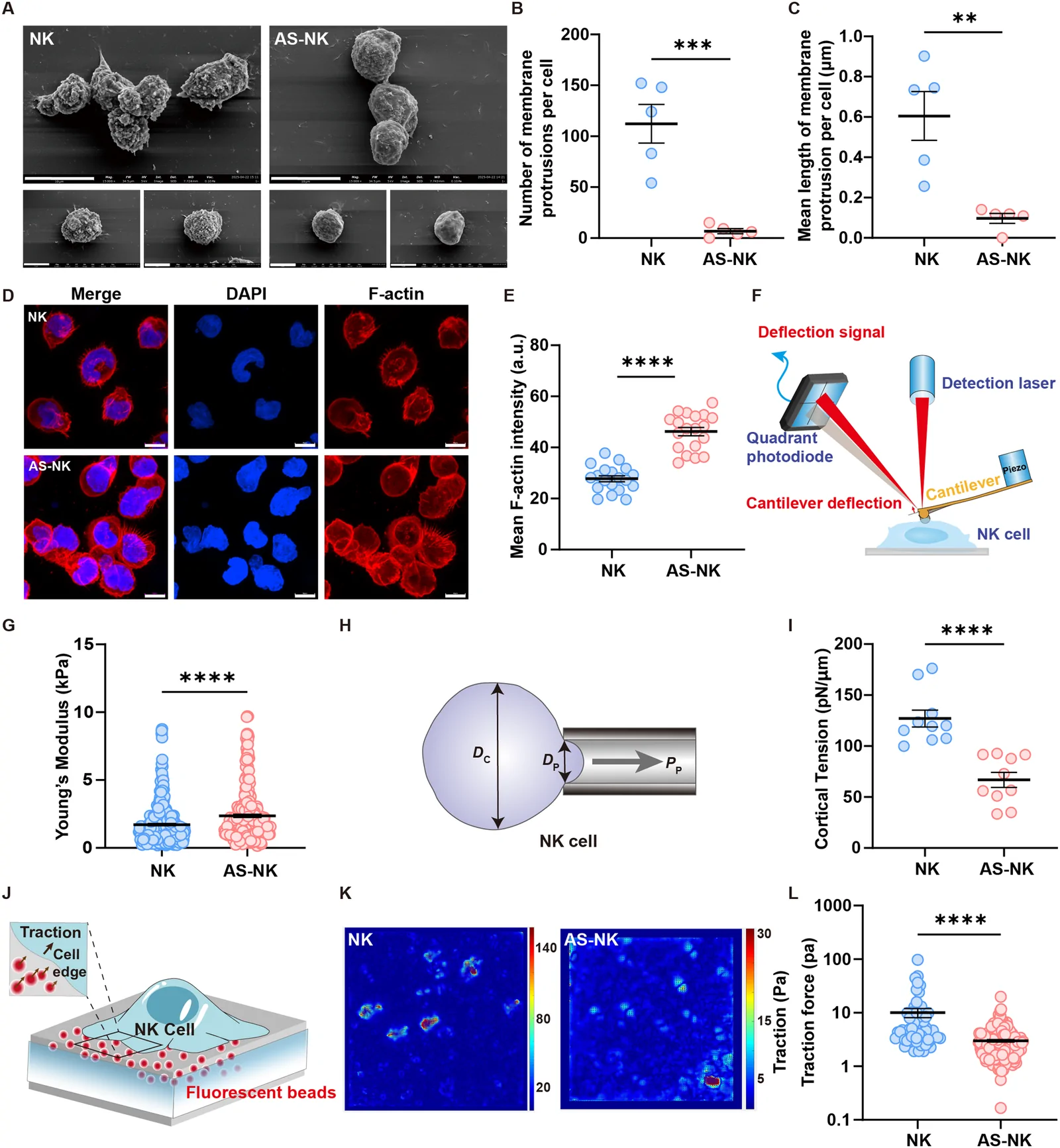
Ovarian cancer ascites remodels the mechanophenotypes of NK cells. **(A–C)** Representative SEM images **(A)** and statistical results of the mean number **(B)** and length **(C)** of the surface membrane protrusions of NK and AS-NK cells [**(B, C)** *n* = 5 independent SEM images per group, Scale bars, 10 µm (top) and 5 µm (bottom)]. **(D, E)** Representative immunofluorescence images **(D)** and statistical results **(E)** of F-actin expression in NK and AS-NK (**(E)** *n* = 19 cells per group, scale bars = 10 μm). **(F, G)** Schematic **(F)** and statistical results **(G)** of AFM measuring cellular stiffness (Young’s modulus) of NK and AS-NK cells [**(G)** NK, *n* = 339 cells; AS-NK, *n* = 283 cells]. **(H, I)** Schematic **(H)** and statistical results **(I)** of cortical tension measurements of NK and AS-NK cells [**(I)** n = 10 cells per group]. **(J–L)** Schematic **(J)**, representative traction force microscopy heat maps **(K)** and statistical results **(L)** of traction forces from NK and AS-NK cells [**(L)** NK, *n* = 61 cells; AS-NK, *n* = 188 cells]. Data are presented as mean ± s.e.m. **, *** and **** refer to *p* < 0.01, *p* < 0.001 and *p* < 0.0001, respectively in unpaired *t*-tests.

We next compared the cortical tension, a key parameter reflecting the mechanical resistance at the cell periphery governed by membrane-actin cortex linkage, between untreated and ascites-conditioned NK cells. Intriguingly, despite their increased global stiffness, ascites-conditioned cells exhibited a significant reduction in cortical tension, as measured by micropipette aspiration (**Figures 2H, I**). This specific weakening of the cortical tension was critically important because the cell cortex provided the structural foundation for force transmission. Using traction force microscopy (TFM), we found that the impaired cortex directly compromised the cell’s ability to apply traction forces against a substrate (calculated from the displacement of fluorescent beads in an elastic substrate; **Figures 2J–L**), a mechanical output essential for immune synapse stabilization and cytotoxic killing (24). In line with the reduced traction force, AS-NK cells also exhibited significantly impaired migration in the transwell assay (**Supplementary Figure 2**).

Given the elevated F-actin abundance in AS-NK cells, together with the concomitant reductions in cortical tension and traction force, we next examined whether the spatial distribution of F-actin was also altered, which could explain the functional dissociation between actin content and mechanical output. Using radial distribution analysis based on confocal fluorescence images, each single-cell region of interest (ROI) was divided into 10 radial zones from the cell center to the edge (**Figure 3A**), allowing quantitative assessment of F-actin distribution. Three complementary parameters were quantified: intensity-weighted radial position, peripheral/central intensity ratio, and edge/inner-cell intensity ratio (**Figure 3B**). Radial distribution analysis revealed a clear redistribution of F-actin in AS-NK cells (**Figure 3C**). Compared with untreated NK cells, AS-NK cells exhibited significantly reduced intensity-weighted radial position, peripheral/central intensity ratio, and edge/inner-cell intensity ratio (**Figures 3D–F**), indicating that F-actin was less efficiently enriched at the cell cortex and relatively redistributed toward inner cellular regions.

**Figure 3:**
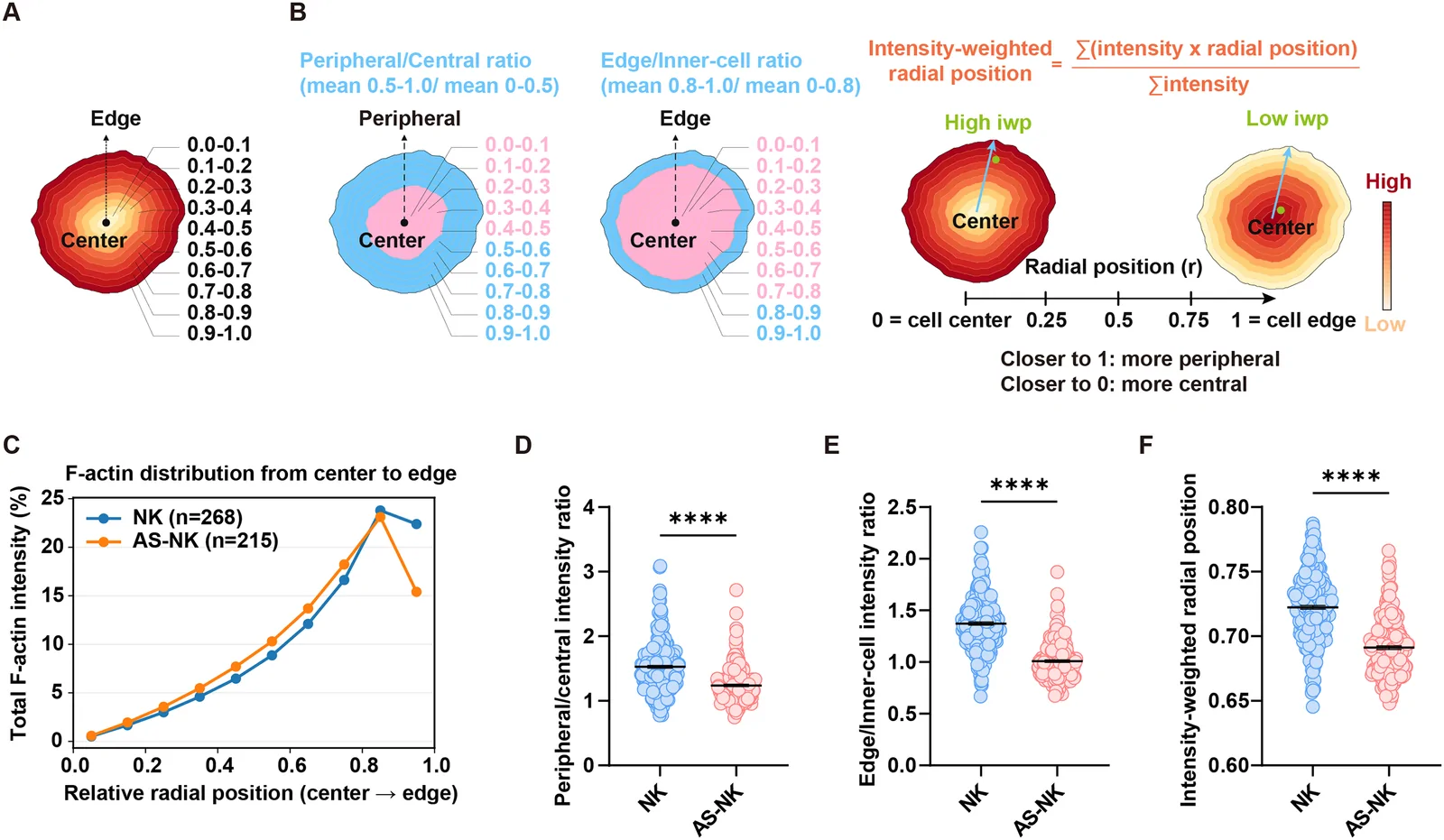
Radial binning analysis reveals reduced cortical enrichment of F-actin in ascites-treated NK cells. **(A)** Schematic illustration of F-actin radial binning analysis. Each single-cell ROI was divided into 10 radial zones from the cell center to the edge. The relative radial position was normalized from 0 to 1, where 0 represents the cell center and 1 represents the cell edge. **(B)** Schematic illustration of quantitative parameters used for single-cell F-actin spatial analysis, including the peripheral/central intensity ratio, edge/inner-cell intensity ratio, and intensity-weighted radial position. The peripheral/central intensity ratio was calculated as the mean F-actin intensity in the peripheral region (0.5–1.0) divided by that in the central region (0–0.5). The edge/inner-cell intensity ratio was calculated as the mean F-actin intensity in the edge region (0.8–1.0) divided by that in the inner-cell region (0–0.8). The intensity-weighted radial position represents the intensity-weighted average radial position of F-actin signals, with higher values indicating a distribution closer to the cell edge. **(C)** Radial percentage distribution of total F-actin intensity from the cell center to the edge in NK and AS-NK cells. **(D–F)** Quantification of peripheral/central intensity ratio **(D)**, edge/inner-cell intensity ratio **(E)** and intensity-weighted radial position **(F)**. Each dot represents one cell. NK, *n* = 268 cells; AS-NK, *n* = 215 cells. Data are presented as mean ± s.e.m. Statistical significance was determined using a two-sided Mann–Whitney U test. **** *p* < 0.0001.

Collectively, our data indicate that ascites remodels the NK cell cytoskeleton into a dysfunctional state of “enhanced but disabled actin polymerization”, where total F-actin content is elevated while its cortical enrichment and spatial organization are perturbed, failing to assemble a mechanically competent cortex capable of producing the forces necessary for effective target cell engagement and killing.

### Ovarian cancer ascites impairs NK cell killing dynamics

Given the observed alterations in cytoskeletal mechanics and traction force, we next asked whether ascites conditioning functionally impairs the dynamic process of NK cell-mediated killing. To directly visualize this process, we performed live-cell imaging to track interactions between NK cells and SKOV3 target cells in real time for 8 hours. The NK cell-mediated killing of a SKOV3 cell proceeded through a distinct sequence: it initiated with target recognition and immune synapse formation, followed by cytotoxic granule release that triggers apoptotic contraction of the target cell, and culminated in membrane rupture with propidium iodide (PI) influx, marking irreversible apoptotic death (**Figure 4A**, **Supplementary Figure 3**). All NK cells that formed a discernible contact with a target cell were categorized into two distinct functional outcomes based on the fate of the target cell: successful killing versus sustained but non-cytolytic contact (**Figures 4B, C**). Compared with untreated NK cells, AS-NK cells exhibited a significantly lower percentage of successful killing events (63.92% ± 5.62% vs. 38.08% ± 3.05%; **Figure 4B**) and a corresponding increase in sustained non-cytolytic contacts (36.08% ± 5.62% vs. 61.92% ± 3.05%; **Figure 4C**), indicating impaired post-synaptic execution of cytotoxicity. Consistently, even in successful killing events, the time interval from immune synapse formation to target cell death *(*Δ*t*) was significantly prolonged in AS-NK cells (**Figure 4D**). Together, these real-time data demonstrate that ascites conditioning impairs cytolytic execution within stable conjugates, as evidenced by increased sustained non-cytolytic contacts and prolonged Δ*t*, thereby diminishing overall killing efficiency.

**Figure 4:**
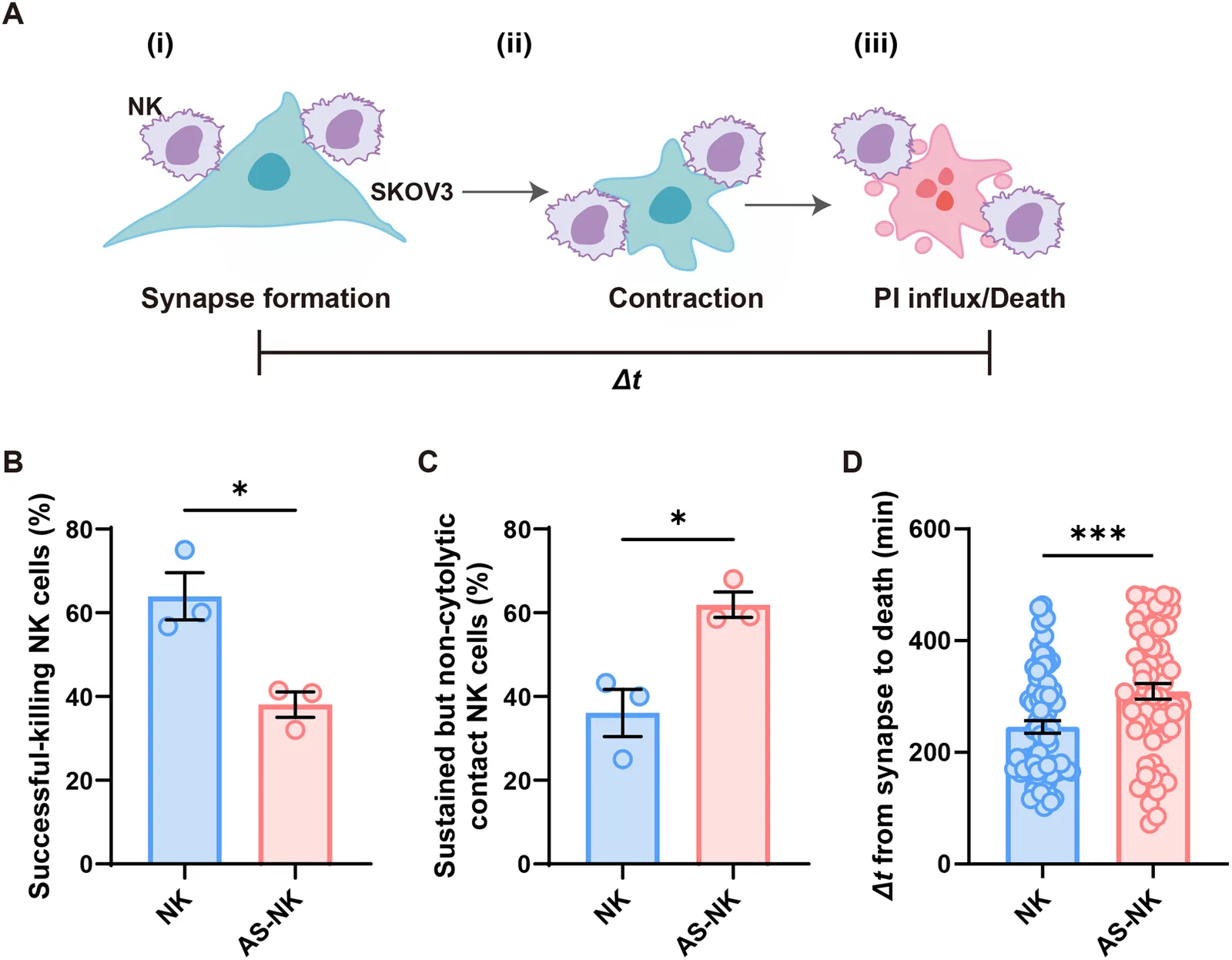
Ascites treatment impairs NK-cell killing dynamics. **(A)** Schematic depicting the NK cell killing a SKOV3 cell. **(B–D)** Comparisons of the percentage of successful-killing NK cells **(B)**, the percentage of sustained but non-cytolytic contact NK cells **(C)**, and the time from synapse formation to target-cell death **(D)** between NK and AS-NK groups. [**(B, C)** *n* = 3 independent experiments; **(D)** NK, *n* = 75 cells; AS-NK, *n* = 65 cells] Data are presented as mean ± s.e.m. * and *** refer to *p* < 0.05 and *p* < 0.001, respectively, in unpaired *t*-tests.

### Dysregulated cytoskeletal transcriptome in ascites NK cells converges on CDC42 pathway inhibition

Building upon our functional evidence that ascites disrupted NK cell cytoskeletal mechanics and cytotoxicity, we hypothesized that these defects would be reflected in the transcriptomic landscape. To further investigate the molecular basis of NK cell dysfunction in ascites, we reanalyzed the bulk RNA-sequencing data from our previous study, where the transcriptomes of NK cells isolated from ovarian cancer ascites were compared to those derived from cord blood mononuclear cells (control) (7). Differential expression analysis was performed using DESeq2, with *p*-values adjusted by the Benjamini−Hochberg procedure (FDR < 0.01). Applying a threshold of |log_2_FC| ≥ 1 and q < 0.01, we identified 6548 differentially expressed genes (DEGs), of which 4283 were up-regulated and 2265 were down-regulated in ovarian cancer ascites-isolated NK cells relative to the control NK cells (**Figure 5A**).

**Figure 5:**
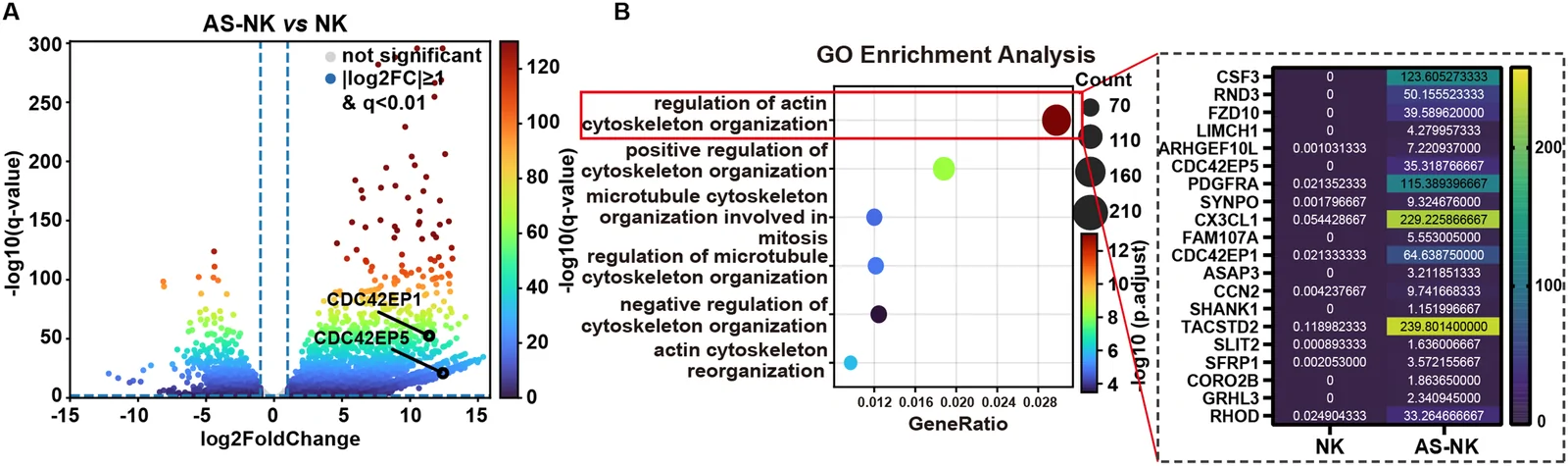
Transcriptomic profiling of NK cells isolated from ovarian cancer ascites versus control NK cells derived from cord blood mononuclear cells. **(A)** Volcano plot of differentially expressed genes (DEGs). **(B)** Gene Ontology (GO) enrichment analysis of the DEGs. For the most significantly enriched term, the associated top 20 dysregulated genes were listed alongside their expression levels.

To directly link these transcriptional changes to the observed functional phenotypes, we performed a targeted enrichment analysis focusing on predefined Gene Ontology terms related to cytoskeletal regulation, confirming a significant enrichment of the DEGs within these categories (**Figure 5B**). Strikingly among the most enriched pathway (GO:0032956, regulation of actin cytoskeleton organization), further analysis of the top 20 most dysregulated genes revealed that multiple regulators of small GTPase signaling underwent a striking transition from near-undetectable levels in control NK cells to significant expression in ascites-isolated NK cells (**Figure 5B**), including not only Rho family GTPases (such as RND3 and RHOD), but their related proteins (such as ARHGEF10L, CDC42EP1 and CDC42EP5). This coordinated *de novo* induction strongly suggested that ovarian cancer ascites may remodel NK cell cytoskeletal mechanics by rewiring small GTPase-dependent pathways. Notably, CDC42EP1 and CDC42EP5 are established negative regulators of CDC42 activity (25). Their striking transition from near-undetectable to significant expression in ascites-infiltrated NK cells directly implied that CDC42 functional output was suppressed in this microenvironment. Among the broadly dysregulated GTPase-related molecules, the specific induction of these CDC42-targeting inhibitors positioned CDC42 as a key signaling hub likely responsible for the mechanical and functional impairment of NK cells in ovarian cancer ascites.

### Bidirectional pharmacological modulation of CDC42 activity establishes traction force as a mechanobiomarker of NK cell cytotoxicity

To directly assess how ovarian cancer ascites affects CDC42 at the protein level, we performed immunofluorescence staining to evaluate both its total expression and activation state in NK cells. Although CDC42 itself was not among the top 20 dysregulated genes, its protein level and, more importantly, its activation were significantly reduced following ascites conditioning (**Figures 6A, B**, **Supplementary Figure 4**). This decrease in functional CDC42 aligns with the concurrent upregulation of its negative regulators, CDC42EP1 and CDC42EP5, in ascites-isolated NK cells.

**Figure 6:**
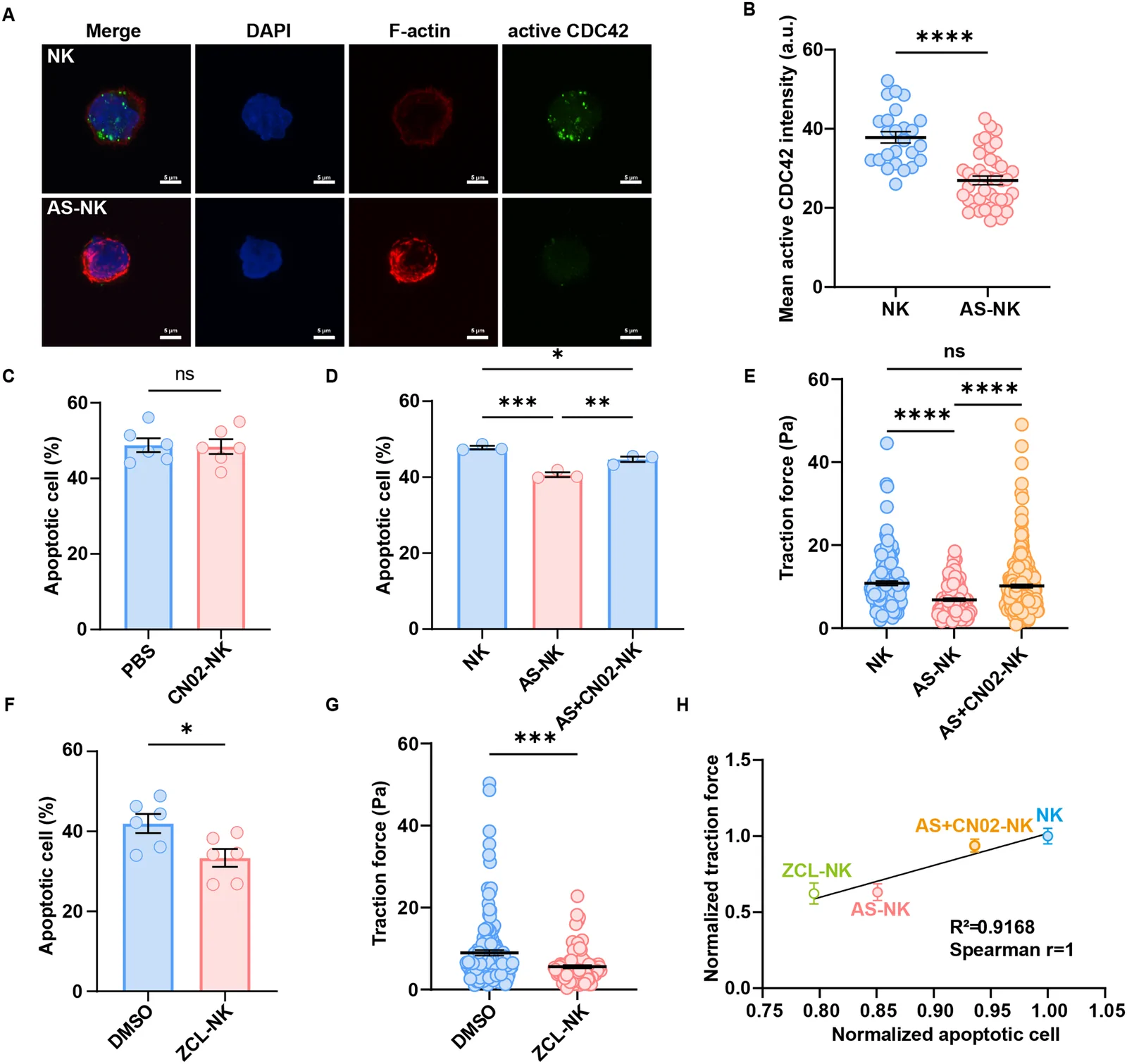
Pharmacological modulation of CDC42 activity bidirectionally regulates NK cell cytotoxicity and traction force. **(A, B)** Representative immunofluorescence images **(A)** and statistical results **(B)** of active CDC42 in NK and AS-NK (scale bars = 5 μm; NK, *n* = 25; AS-NK, *n* = 40). **(C, D)** Comparisons of percentages of apoptotic SKOV3 cells after 5 h co-culture with NK or CN02-NK **(C)**, and with NK, AS-NK, or AS+CN02-NK **(D)**. **(E)** Comparisons of traction force of NK, AS-NK, and AS+CN02-NK cells (NK, *n* = 138; AS-NK, *n* = 101; AS+CN02-NK, *n* = 252). **(F, G)** Comparisons of percentages of apoptotic SKOV3 cells after 5 h co-culture with NK or ZCL-NK cells **(F)** and the traction forces of NK or ZCL-NK cells **(G)** (NK, *n* = 146; ZCL-NK, *n* = 104). **(H)** Correlation analysis of normalized NK cell traction force and normalized SKOV3 cell apoptosis rate (*n* = 4; linear fit *R*^2^ = 0.9168; Spearman *r* = 1). Data are presented as mean ± s.e.m. ns, *, **, *** and **** refer to no significant difference, *p* < 0.05, *p* < 0.01, *p* < 0.001 and *p* < 0.0001, respectively in unpaired *t*-tests **(B, C, F, G)** or one-way ANOVA **(D, E)**.

We therefore asked whether pharmacologically restoring CDC42 activity could rescue NK cell dysfunction. Using the NK-SKOV3 co-culture cytotoxicity assay, we found that pretreatment with CN02, a specific CDC42 activator, did not further augment the killing capacity of untreated NK cells (**Figure 6C**, **Supplementary Figure 5A**). Strikingly, however, CN02 treatment significantly reversed the suppressed tumor-killing capacity of ascites-conditioned NK cells, though not fully restoring it to the level of untreated cells (**Figure 6D**, **Supplementary Figure 5B**). Mechanistically, traction force microscopy revealed that CN02 treatment significantly restored the compromised traction force in these cells, paralleling the recovery in cytotoxicity (**Figure 6E**, **Supplementary Figure 6A**). This coordinated restoration of both mechanical output and killing function suggests that CDC42 regulates NK cell anti-tumor efficacy, at least in part, through modulating traction force generation. Conversely, to explore whether CDC42 inhibition could recapitulate the ascites-induced phenotype, we treated otherwise competent NK cells with the specific CDC42 inhibitor ZCL278. ZCL278 pretreatment significantly attenuated tumor-killing function (**Figure 6F**, **Supplementary Figure 5C**) and markedly reduced traction force (**Figure 6G**, **Supplementary Figure 6B**), mirroring the effects of ascites exposure. This parallel decline in both mechanical output and cytotoxic efficacy was further supported by live-cell imaging, which showed that ZCL278 treatment reduced successful-killing events, increased sustained non-cytolytic contacts, and prolonged the time from synapse formation to target-cell death (**Supplementary Figures 7A–C**). In contrast, CN02 treatment did not significantly alter these killing-dynamic parameters in otherwise competent NK cells (**Supplementary Figures 7D–F**). In addition, ZCL278 treatment reduced perforin expression and IFN-γ secretion, whereas GZMB and CD107a expression remained unchanged—a pattern similar to that observed in AS-NK cells (**Supplementary Figure 8**). At the structural level (**Supplementary Figure 9A**), ZCL278 treatment also reduced F-actin intensity (**Supplementary Figure 9B**) and diminished membrane protrusion formation (**Supplementary Figures 9C, D**), whereas CN02 treatment increased F-actin intensity (**Supplementary Figure 9E**) without significantly altering membrane protrusions (**Supplementary Figures 9F, G**). Together, these findings indicate that CDC42 inhibition largely phenocopies the functional, mechanical, and structural alterations induced by ascites, further supporting CDC42 as a key node mediating ascites-induced NK cell dysfunction.

To further evaluate the mechanistic link between cytoskeletal mechanics and NK cell cytotoxicity, we performed correlation analysis across aforementioned four experimental conditions, ZCL278-pretreated (ZCL-NK), untreated (NK), ascites-conditioned (AS-NK), and ascites-conditioned together with CN02 treatment (AS+CN02-NK). Both linear regression and Spearman rank correlation revealed a strong and significant positive correlation between NK cell killing capacity and traction force generation (linear fit *R*² = 0.9168; Spearman *r* = 1) (**Figure 6H**). This near-perfect association reinforces that traction force is a robust mechanical readout tightly coupled to NK cell effector function. More importantly, it highlights the potential of using traction force as a mechanobiomarker for evaluating and predicting NK cell-mediated anti-tumor immunity, and suggests that restoring mechanical competence could serve as a functional endpoint for immunotherapeutic interventions in the suppressive tumor microenvironment. Conversely, targeted attenuation of NK cell mechanics via CDC42 inhibition may offer a strategy to curb hyperactive cytotoxicity in autoimmune settings.

### Proteinaceous components in ovarian cancer patient-derived ascites contribute to NK-cell cytoskeletal and mechanical dysfunction

To further investigate how ovarian cancer patient-derived ascites impairs the mechanophenotypes of NK cells, we prepared protein-depleted ascites using 3 kDa cutoff centrifugal filters, which reduced the total protein content by approximately 91.6%. NK cells were then treated with intact ascites (AS-NK) or protein-depleted ascites (ΔPr-NK). Protein depletion largely attenuated the ascites-induced mechanical and cytoskeletal defects, restoring traction force (**Figures 7A, B**), membrane protrusion formation (**Figures 7C–E**), F-actin intensity (**Figure 7F**), and active CDC42 intensity (**Figure 7G**) to levels comparable to those of untreated NK cells. These findings in the first step indicate that the proteinaceous fraction of ovarian cancer ascites is required for the major cytoskeletal and mechanical alterations observed in ascites-treated NK cells.

**Figure 7:**
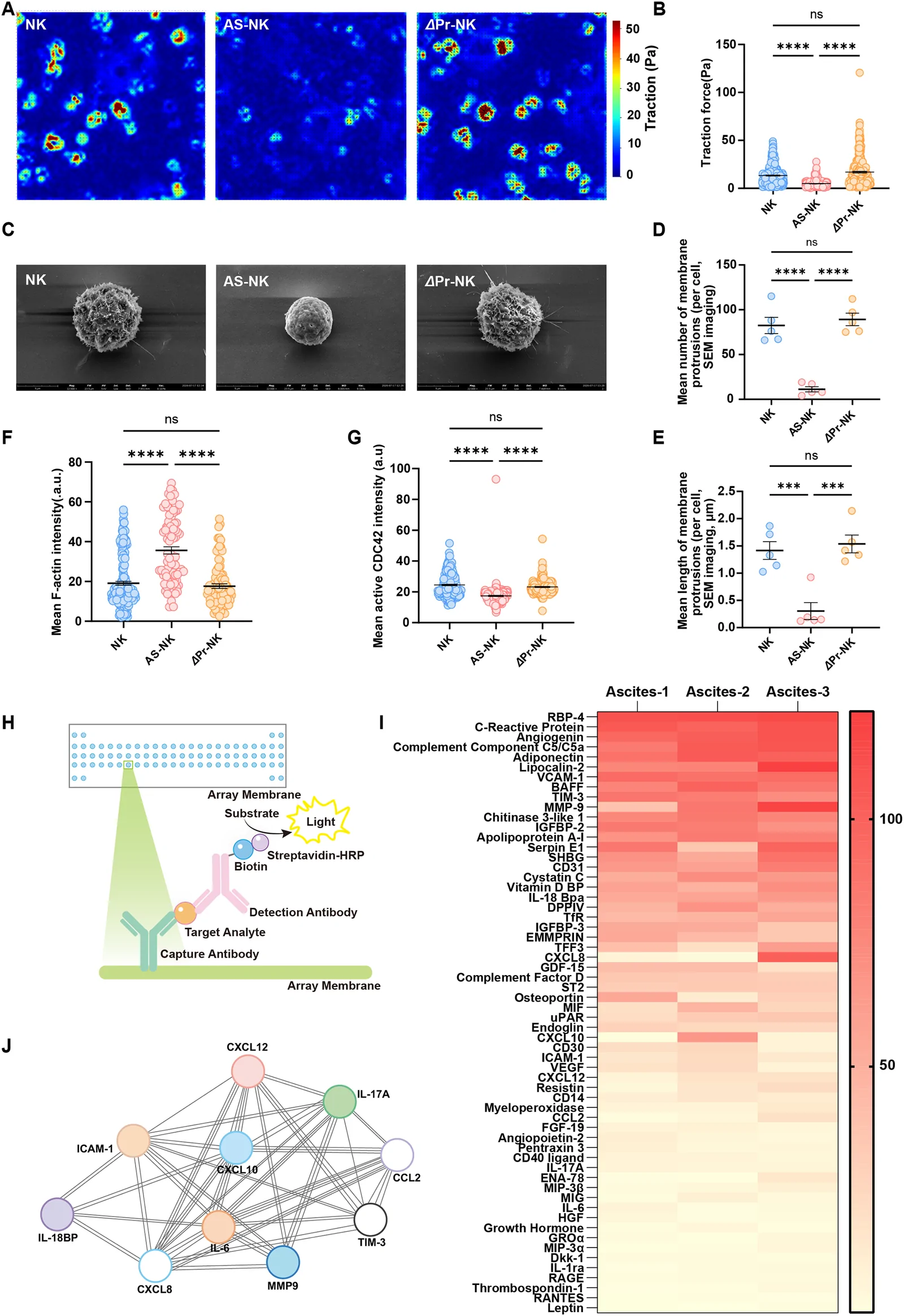
Proteinaceous components of ovarian cancer ascites contribute to NK-cell cytoskeletal and mechanical dysfunction. **(A)** Representative traction force microscopy heat maps of untreated NK cells (NK), intact ascites-treated NK cells (AS- NK), and protein-depleted ascites-treated NK cells (ΔPr-NK). **(B)** Quantification of traction force in NK, AS-NK, and ΔPr-NK cells (NK, *n* = 357 cells; AS-NK, *n* = 312 cells; ΔPr-NK, *n* = 337 cells). **(C)** Representative scanning electron microscopy (SEM) images showing membrane morphology and protrusions of NK, AS-NK, and ΔPr-NK cells. **(D, E)** Quantification of the mean number **(D)** and mean length **(E)** of membrane protrusions (*n* = 5 independent SEM images per group). **(F)** Quantification of mean F-actin intensity in NK, AS-NK, and ΔPr-NK cells (NK, *n* = 181 cells; AS-NK, *n* = 86 cells; ΔPr-NK, *n* = 91 cells). **(G)** Quantification of mean active CDC42 intensity in NK, AS-NK, and ΔPr-NK cells (NK, *n* = 241 cells; AS-NK, *n* = 237 cells; ΔPr-NK, *n* = 212 cells). **(H)** Schematic illustration of the solid-phase antibody array used to profile soluble protein components in ovarian cancer ascites. Ascites analytes were captured by membrane-bound antibodies and detected using biotinylated detection antibodies and streptavidin-HRP. **(I)** Heatmap showing the relative abundance of soluble factors detected in ascites samples from three independent patients (Ascites-1, Ascites-2, and Ascites-3). The color scale indicates relative signal intensity. **(J)** Protein-protein interaction (PPI) network of ten NK-cell function-associated factors detected in ascites, constructed using the STRING database. Data are presented as mean ± s.e.m. ns, **, *** and **** indicate no significant difference, *p* < 0.01, *p* < 0.001 and *p* < 0.0001, respectively, as determined by Kruskal-Wallis tests.

Having established that the proteinaceous fraction of ascites is required for the cytoskeletal and mechanical defects in NK cells, we next sought to identify candidate protein components that might explain these observations. We profiled the soluble proteomic landscape of ovarian cancer ascites using an antibody array targeting 105 factors (**Figure 7H**). Analysis of ascites from three independent patients (**Table S1**) revealed a highly concordant profile, with approximately 60 factors detected in each sample (**Figure 7I**). These factors included three major categories: pro-inflammatory or chemotactic factors (*e.g.*, IL-6, IL-8, CCL2), immunoregulators (*e.g.*, TIM-3), and angiogenic mediators (*e.g.*, VEGF, Angiopoietin-2) (**Figure 7I**, **Supplementary Figure 10A**). Based on existing literature, several detected factors, including TIM-3 (HAVCR2), MMP-9, IL-18BP, IL-6, ICAM-1, and IL-17A, are implicated in regulating NK cell activation, whereas IP-10 (CXCL10), IL-8 (CXCL8), SDF-1α (CXCL12), and MCP-1 (CCL2) are associated with NK cell migration (26–34).

To investigate the potential cooperative roles of these ten key factors in modulating NK cell anti-tumor immunity, we constructed a protein-protein interaction (PPI) network using the STRING database (**Figure 7J**). The analysis revealed that these molecules do not function in isolation but form a densely interconnected, functionally intertwined regulatory network. Notably, several chemokines (*e.g.*, IL-8/CXCL8, IP-10/CXCL10, SDF-1α/CXCL12) and inflammatory factors (*e.g.*, IL-6, IL-17A) served as highly connected hubs within the network (**Figure 7J**). This strong interconnectivity suggests that these ascites-derived factors may coregulate NK cell function through shared or convergent downstream signaling pathways, rather than exerting isolated effects. Interestingly, four of the ten key factors, specifically the chemokines IP-10 (CXCL10), SDF-1α (CXCL12), and MCP-1 (CCL2), directly regulate actin cytoskeleton dynamics (**Supplementary Figure 10B**). Moreover, other identified molecules, including ICAM-1 and TIM-3, have also been reported to influence cytoskeletal organization and remodeling (**Supplementary Figures 10C, D**). These findings, together with our protein-depletion experiments, suggest that the proteinaceous components within ovarian cancer ascites, particularly those converging on actin cytoskeleton regulation, may contribute to the cytoskeletal disorganization and mechanical dysfunction observed in ascites-treated NK cells.

## Discussion

In this study, we identify a previously unrecognized mechanism by which the ovarian cancer ascites microenvironment suppresses NK cell cytotoxicity: chronic ascites exposure induces CDC42EP1/EP5 expression, suppressing CDC42 activity and leading to cytoskeletal disorganization, impaired force generation, and defective immune synapse formation (**Figure 8**). Integrating biophysical, imaging, transcriptomic, and pharmacological approaches, we reveal a paradoxical cytoskeletal state in ascites-conditioned NK cells where increased F-actin fails to generate productive force, resulting in reduced cortical tension and traction force (**Figure 8**). We further establish CDC42 as a central mechanistic hub: its suppression, driven by *de novo* induction of CDC42EP1/EP5, underlies these cytoskeletal defects, and bidirectional modulation of CDC42 activity reciprocally regulates traction force and cytotoxicity (**Figure 8**). These findings introduce traction force as a mechanobiomarker of NK cell competence and position CDC42 as a therapeutically actionable target for restoring anti-tumor immunity in immunosuppressive microenvironments.

**Figure 8:**
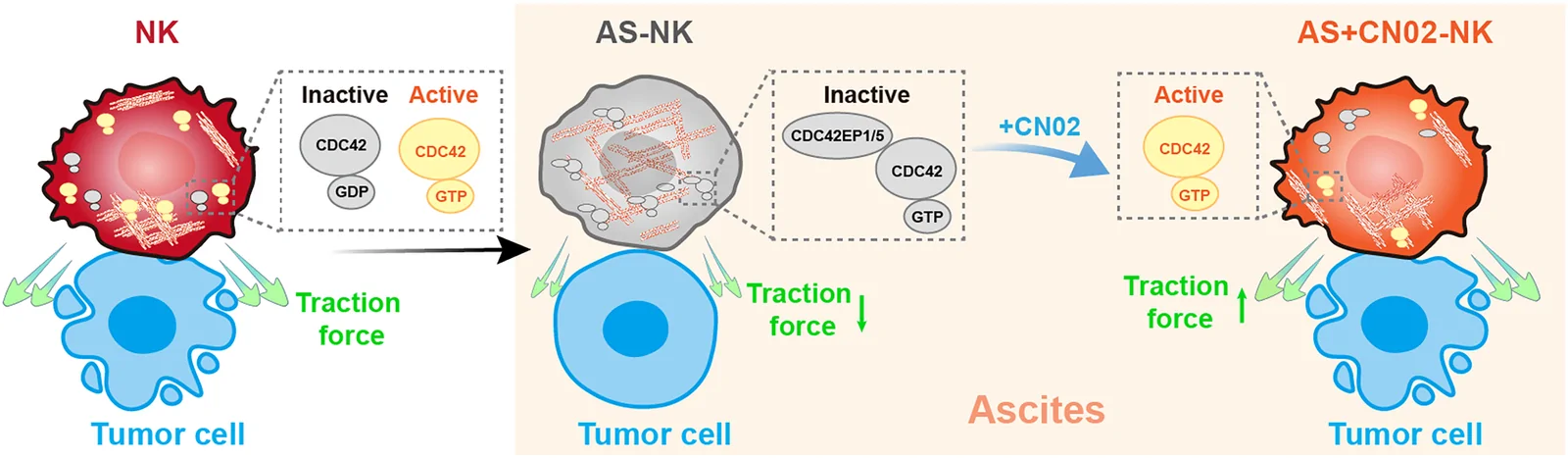
Schematic model depicting the regulation of NK cell cytotoxicity by CDC42-dependent traction force. Left: Untreated NK cells display normal CDC42 activity, which supports robust traction force generation and efficient killing of tumor cells. Middle: Upon exposure to ovarian cancer ascites, the upregulation of CDC42 negative regulators (CDC42EP1 and CDC42EP5) suppresses CDC42 activity, resulting in diminished traction force and impaired tumor cell killing. Right: Pharmacological activation of CDC42 with the specific activator CN02 restores CDC42 activity and traction force, thereby rescuing NK cell-mediated cytotoxicity.

The observation that ascites-treated NK cells accumulate more F-actin yet generate less force challenges the intuitive assumption that increased actin polymerization necessarily equates to enhanced cytoskeletal function. This apparent paradox is reconciled by our finding that mechanical performance is governed by functional cytoskeletal output, not actin abundance per se. Indeed, the elevated F-actin content, coupled with reduced cortical tension and impaired traction force, points to a failure in assembling F-actin into mechanically competent structures capable of force transmission. Collectively, our data suggest that ascites induces a state of “disabled actin polymerization”, where actin is polymerized but fails to be organized into force-generating networks. This interpretation aligns with recent studies in T cells showing that cytoskeletal integrity and proper architecture, rather than total actin content, determine immune synapse maturation and signaling (35–37). We acknowledge that direct visualization of cortical actin architecture by super-resolution microscopy would further strengthen this interpretation, and that actin crosslinkers (*e.g.*, Filamin A, α-actinin) and membrane-cytoskeleton linker proteins (*e.g.*, ERM family), which are critical for cortical integrity and force transmission, were not examined in this study (38, 39). Future work combining super-resolution imaging with targeted protein analysis will be needed to fully elucidate the structural basis of this dysfunction.

The ascites microenvironment is a complex cocktail of soluble factors (IL-6, IL-17A, CXCL10, etc.). Our finding that multiple GTPase-related molecules exhibit *de novo* expression suggests that ascites actively rewires the entire small GTPase signaling network, rather than simply suppressing individual nodes. Within this reprogrammed network, the specific induction of CDC42EP1 and CDC42EP5 is particularly significant. These proteins are established negative regulators of CDC42 signaling (25, 40). Seminal studies on the Borg family (to which CDC42EPs belong) demonstrated that they are dynamically engaged upon CDC42 activation, but that proper CDC42 cycling, the transition between active and inactive states, is required for their function (25). Chronic or constitutive activation of CDC42 disrupts Borg-mediated cytoskeletal organization, suggesting that these proteins operate as part of a feedback module that fine-tunes CDC42 output (40). In ascites-conditioned NK cells, however, this finely tuned mechanism appears to be pathologically altered. CDC42EP1 and CDC42EP5 are not transiently induced but constitutively expressed at high levels, effectively transforming them from temporary “off switches” into permanently engaged brakes. This creates a state where CDC42 is chronically suppressed, unable to sustain the dynamic activity required for cytoskeletal remodeling and force generation. This model explains the partial rescue observed with CN02 treatment. While CN02 can forcibly activate CDC42, the persistently high levels of CDC42EP1 and CDC42EP5 rapidly bind and inactivate newly activated CDC42 molecules. Thus, the brake remains engaged even when the accelerator is pressed. Additional layers of suppression, such as altered integrin signaling or metabolic dysfunction, may further contribute to the incomplete functional recovery (41, 42).

The near-perfect correlation between traction force and killing capacity across four distinct experimental conditions (untreated, ascites-treated, CN02-rescued, ZCL278-inhibited) establishes traction force as a robust mechanobiomarker of NK cell functional competence. This finding has significant translational implications: traction force measurements could serve as a functional readout for screening immunomodulatory compounds, evaluating patient-derived NK cell fitness, or monitoring response to immunotherapy. Unlike traditional cytotoxicity assays that measure end-point killing, traction force provides a quantitative, real-time, and mechanism-based parameter that reflects the underlying cytoskeletal health of the effector cell. Moreover, our bidirectional manipulation of CDC42 opens two distinct therapeutic avenues: CDC42 activation to restore NK cell function in immunosuppressive tumor microenvironments (*e.g.*, ovarian cancer ascites), and CDC42 inhibition to dampen hyperactive NK cell responses in autoimmune or inflammatory conditions where excessive cytotoxicity contributes to tissue damage. The fact that CN02 treatment did not further enhance healthy NK cell killing but specifically rescued suppressed cells suggests that this approach may selectively target dysfunctional cells while sparing normal immune homeostasis-a desirable feature for immunotherapy.

Several limitations of this study should be acknowledged. First, our mechanistic experiments were performed using the NK-92MI cell line. Further validation in primary human NK cells from ovarian cancer patients is necessary to confirm clinical relevance. Second, the correlation analysis, though strong, was based on four experimental conditions; expanding the dataset with additional perturbations or patient samples would further strengthen the statistical power. Third, the precise molecular mechanisms by which ascites induces CDC42EP1/5 expression remain to be elucidated. Whether this occurs through direct transcriptional regulation by ascites factors (*e.g.*, IL-6, CXCL10) or via indirect epigenetic reprogramming warrants further investigation. Fourth, while we focused on CDC42, other Rho GTPases (*e.g.*, Rac1, RhoA) and their regulators likely contribute to the observed mechanophenotypes and should be explored in future studies. Fifth, the cytotoxicity assays in this study were performed using only the SKOV3 ovarian cancer cell line as target cells. While this well-established cell line provided a stable and reproducible system for assessing NK cell function, we acknowledge that ovarian cancer encompasses multiple histological subtypes that may differ in their susceptibility to NK-cell-mediated killing (43). Whether the ascites-induced NK cell dysfunction is conserved across different ovarian cancer subtypes and target cell models remains to be determined.

In conclusion, this study uncovers a mechano-immunological paradigm in which the ovarian cancer ascites microenvironment induces NK cell dysfunction by suppressing CDC42 mediated force generation, leading to defective immune synapse maturation and impaired cytotoxicity. The identification of traction force as a mechanobiomarker and CDC42 as a druggable target opens new opportunities for mechano-immunotherapy, an approach that restores immune cell function by targeting both their physical and biochemical properties. Future work should focus on translating these findings to primary human NK cells, developing clinically applicable methods to measure traction force, and evaluating CDC42-targeted interventions in preclinical models of ovarian cancer and autoimmune diseases.

## Materials and methods

### Reagents and tools table

**Table:** 

Reagent/resource	Reference or source	Identifier or catalog number
Experimental Models		
Human ovarian cancer cell line SKOV3	Kindly provided by Prof. Xing Zhao, Guizhou Medical University	N/A
Human NK cell line NK-92MI	Kindly provided by Prof. Xing Zhao, Guizhou Medical University	N/A
Human malignant ovarian cancer ascites samples	Human patients	Ethics approval No. 2023-134
Recombinant DNA		
N/A	N/A	N/A
Antibodies		
CD45 antibody	BioLegend, USA	Cat. #368504
Perforin antibody	BD Biosciences, USA	Cat. #567721
Granzyme B antibody	BioLegend, USA	Cat. #372204
CDC42 antibody	Santa Cruz Biotechnology, USA	Cat. #sc-8401
Active CDC42 antibody	NewEast Biosciences, USA	Cat. #26905
CoraLite^®^488-Conjugated AffiniPure Goat Anti-Mouse IgG (H+L)	Proteintech, Wuhan, China	Cat. #SA00013-1
Oligonucleotides and other sequence-based reagents		
N/A	N/A	N/A
Chemicals, enzymes and other reagents		
Fetal bovine serum (FBS)	Gemini Bio, USA	Cat. #900-108
Penicillin-streptomycin (100×)	Solarbio, Beijing, China	Cat. #P1400
Horse serum	Solarbio, Beijing, China	Cat. #S9050
β-Mercaptoethanol	Invitrogen/Gibco, USA	Cat. #21985023
Inositol	Sigma-Aldrich, USA	Cat. #I5125
Folic acid	Sigma-Aldrich, USA	Cat. #F8758
CN02	Cytoskeleton, USA	Cat. #CN02
ZCL278	MedChemExpress, USA	Cat. #HY-13963
Annexin V-FITC/PI apoptosis detection reagent	BD Biosciences, USA	Cat. #556547
BD CBA Human Soluble Protein Flex Set	BD Biosciences, USA	Cat. #560111
Intracellular Fixation & Permeabilization Buffer Set	eBioscience, USA	Cat. #88-8824-00
MICA protein	ABclonal, Wuhan, China	Cat. #ab182709
Proteome Profiler Human XL Cytokine Array Kit	R&D Systems, USA	Cat. #ARY022B
Rhodamine-phalloidin	Solarbio, Beijing, China	Cat. #CA1610
DAPI staining solution	Solarbio, Beijing, China	Cat. #C0065
Software		
FlowJo software	BD/FlowJo LLC, USA	FlowJo V 10.8.1
CBA analysis software	BD Biosciences, USA	N/A
FIJI	National Institutes of Health, USA	https://imagej.net/
JPK SPM Data Processing	Bruker, Germany	Version 6.0
GraphPad Prism 9.5	GraphPad Software, USA	https://www.graphpad.com/
Other		
BD FACSCelesta™ flow cytometer	BD Biosciences, USA	BD FACSCanto II
Laser scanning confocal microscope	Nikon,Japan	NIS-Elements 5.4
Atomic force microscopy	Bruker, Germany	NanoWizard 4
Atomic force microscopy probe	NanoAndMore GmbH, Germany	CSG01

### Cell culture and treatment

The human ovarian cancer cell line (SKOV3) and the NK cell line (NK-92MI) were kindly provided by Prof. Xing Zhao (Guizhou Medical University). Detailed culture conditions have been described previously (7). Briefly, SKOV3 cells were maintained in McCoy’s 5A medium supplemented with 10% fetal bovine serum (FBS; Gemini Bio, USA) and 1% penicillin-streptomycin (Solarbio, Beijing, China). NK-92MI cells were cultured in MEM-α medium containing 12.5% FBS, 12.5% horse serum (Solarbio, Beijing, China), 0.2 mM inositol, 0.02 mM folic acid (Sigma-Aldrich, St. Louis, MO, USA), and 0.1 mM β-mercaptoethanol (Invitrogen, Carlsbad, CA, USA). All cells were incubated at 37 °C in a humidified atmosphere with 5% CO_2_ and passaged every 2~3 days to maintain exponential growth. Malignant ovarian cancer ascites samples were obtained from human patients under a protocol approved by the Ethics Committee of Guizhou Medical University (Approval No. 2023-134). The ascites samples used in this study were derived from patients #5, #6, and #7 in a previously published study (7), all of whom were diagnosed with high-grade serous ovarian carcinoma, FIGO stage IIIC, with peritoneal metastasis (**Table 1**).

**Table 1:** Clinicopathological information of the ascites donors used in this study.

NO.	Age (year)	Pathologic type	FIGO stage	Peritoneal metastasis
#5	55	High-grade serouscarcinoma	IIIC	Yes
#6	69	High-grade serouscarcinoma	IIIC	Yes
#7	44	High-grade serouscarcinoma	IIIC	Yes

(adapted from Ref (7)).

To generate ascites-conditioned NK cells (AS-NK), malignant ovarian cancer ascites samples were centrifuged at 300 × g for 10 min. The supernatant was collected, filtered through a 0.22 μm filter, and either used immediately or stored at -80 °C until use. NK-92MI cells (5~8 × 10^6^) were then cultured in 3 mL MEM-α medium containing 20% ascites (v/v) supernatant for 24 h. For CDC42 activation, NK-92MI cells (3 × 10^6^) were treated for 0.5 h in 3 mL MEM-α medium with either PBS (vehicle control) or the CDC42 activator CN02 (100 ng/mL; Cytoskeleton, Denver, CO, USA; Cat. #CN02). In corresponding figures, the PBS-treated control group was simply labeled as NK, while the activator-treated group was denoted as CN02-NK. For CDC42 inhibition, NK-92MI cells (3 × 10^6^) were treated for 1 h in 3 mL MEM-α medium with either DMSO (vehicle control) or the specific CDC42 inhibitor ZCL278 (50 μM; MedChemExpress, Monmouth Junction, NJ, USA; HY-13963). In corresponding figures, the DMSO-treated control group was labeled as NK for simplicity, and the inhibitor-treated group was denoted as ZCL-NK. To obtain ascites-conditioned NK cells with concomitant CDC42 activation (AS+CN02-NK), NK-92MI cells (3 × 10^6^) were cultured for 24 h in 3 mL MEM-α medium containing both 20% ascites supernatant and CN02 (100 ng/mL).

### Flow cytometry

Percentages of apoptotic SKOV3 cells were assessed at various effector-to-target (E:T) ratios. SKOV3 cells in the logarithmic growth phase were adjusted to a concentration of 1 × 10^6^ cells/mL and seeded into a 48-well plate at 200 μL per well. NK-92MI cells were then added at E:T ratios of 2:1, 4:1, 8:1, and 16:1. After gentle mixing, the co-cultures were incubated at 37 °C with 5% CO_2_ for 5 h. Following incubation, the culture supernatant was collected. The remaining adherent cells were detached by trypsinization, and the digestion was terminated by adding complete culture medium. These detached cells were combined with the cells collected from the supernatant, washed with PBS, and resuspended in 100 μL of 1× Binding Buffer. The cell suspension was stained with 5 μL each of CD45 (BioLegend, Cat. #368504), Annexin V-FITC (BD Biosciences, Cat. #556547), and propidium iodide conjugated to phycoerythrin (PI-PE, BD Biosciences, Cat. #556547) for 15 min at room temperature in the dark. For all subsequent experiments evaluating the effects of CDC42 modulators (CN02, ZCL278), an E:T ratio of 4:1 was selected. Finally, samples were analyzed using a BD FACSCelesta™ flow cytometer. Apoptotic rates were determined using FlowJo 10.

The concentration of IFN-γ in the co-culture supernatant was measured using the BD CBA Human Soluble Protein Flex Set (BD Biosciences, Cat. #560111). A standard curve was generated by serial dilution of the provided standards. The collected supernatants were thoroughly mixed with the capture beads and incubated at room temperature in the dark for 1 h. PE-conjugated detection reagent was then added, followed by another 1 h incubation under the same conditions. After washing and resuspension, samples were acquired on a BD FACSCelesta™ flow cytometer. Data were analyzed using the CBA analysis software, and IFN-γ concentrations were calculated based on the standard curve.

For intracellular staining of perforin and granzyme B, NK-92MI cells from the co-cultures were first stained for the surface marker CD45 as described above. Cells were then fixed with IC Fixation Buffer (eBioscience, Cat. #88-8824-00) for 20 min and permeabilized with Permeabilization Buffer (eBioscience, Cat. #88-8824-00) for 30 min. Subsequently, cells were incubated with perforin antibody (BD Biosciences, Cat. #567721) and granzyme B antibody (BioLegend, Cat. #372204) for 15 min at room temperature in the dark. After washing and resuspension, samples were analyzed on a BD FACSCelesta™ flow cytometer. The percentages of perforin^+^ and granzyme B^+^ cells, or their mean fluorescence intensities (MFI), were determined using FlowJo 10.

### Scanning electron microscopy

NK-92MI cells were fixed with 4% paraformaldehyde. The fixed cells were then seeded onto poly-L-lysine-coated coverslips and incubated at 37 °C for 30-60 min to allow attachment. After washing gently with sterile ddH_2_O, the cells were dehydrated through a graded ethanol series (30%, 50%, 70%, 80%, 90%, and 100% v/v) for 15 min per step. The samples were then dried using a freeze-drying apparatus and examined under a scanning electron microscope (Phenom Nano G2, Phenom Scientific, Shanghai, China). For quantitative analysis, the average number and length of membrane protrusions per cell were measured using FIJI software.

### Atomic force microscopy

For stiffness measurements, NK-92MI cells were seeded onto poly-L-lysine-coated culture dishes and incubated at 37 °C for 30 min to allow attachment. After gentle washing with PBS, the cells were immediately subjected to AFM measurement. Prior to each experiment, the spring constant of the AFM probe (NanoWizard 4, JPK Instruments, Bruker, Germany) was calibrated using the thermal noise method. Force spectroscopy was performed in PBS using contact mode, with the indentation depth set to no more than 10% of the cell height. At least 50 cells per condition were measured. Data were analyzed using the JPK SPM Data Processing software (44).

### Traction force microscopy

Traction forces exerted by NK-92MI cells were measured using TFM. Experiments were performed using a 1 kPa polyacrylamide hydrogel substrate embedded with red fluorescent microspheres. All steps were conducted in the dark to minimize photobleaching. The hydrogel substrate was washed with sterile PBS and then functionalized by incubating with 100 μL of recombinant human MICA protein solution (25 μg/mL; Abclonal, Cat. #ab182709) for 1 h at 37 °C in a 5% CO_2_ incubator. After washing with PBS, 500 μL of NK-92MI cell suspension (8 × 10^5^ cells/mL) was evenly seeded onto the substrate and incubated for 1 h under the same conditions to allow cell adhesion. Phase-contrast images of adherent cells and fluorescence images of the embedded microspheres were acquired. Subsequently, 100 μL of 1 M NaOH solution was added to completely detach the cells. After cell detachment and the relaxation of the hydrogel to its stress-free state, fluorescence images of the microspheres were captured again. The traction force field generated by each cell was reconstructed by analyzing the displacement field of the fluorescent microspheres (45).

### Measurement of cortical tension via micropipette aspiration

The cortical tension of NK-92MI cells was measured using micropipette aspiration. Briefly, glass capillaries were pulled using a micropipette puller, and the tips were forged on a microforge to obtain a smooth, flat opening with an inner diameter of approximately 3-5 μm. The chamber was then filled with 200 μL of DMEM basal medium supplemented with 1% bovine serum albumin (BSA), and 2~4 μL of NK-92MI cell suspension (1 × 10^6^ cells/mL) was gently added. The prepared micropipette was connected to a pressure-controlled hydraulic system. Under an inverted microscope, the following parameters were recorded for each cell: the inner diameter of the micropipette (*D_p_*), the diameter of a freely floating cell (*D*_C_), and the critical aspiration pressure (*P_p_*) required to draw the cell into the pipette until a hemispherical projection matching the pipette inner diameter was formed. Cortical tension (*T*_C_) was then calculated using the Young-Laplace equation, as shown in Equation 1:

(1) $$T_{\text{c}}=\;\frac{P_{\text{p}}}{2(1/R_{\text{p}}\;-\;1/R_{\text{c}})}$$

where *R*_p_ and *R*_c_ are the radii of the pipette and the cell, respectively (46, 47).

### Detection of soluble cytokines in human ovarian cancer ascites samples

Soluble cytokines in ovarian cancer ascites samples (*n* = 3) were profiled using a Proteome Profiler Human XL Cytokine Array Kit (R&D Systems, #ARY022B) following the manufacturer’s instructions. Briefly, array membranes were blocked with Array Buffer 6 for 1 h, then incubated overnight at 2~8 °C with diluted ascites samples. After washing, membranes were incubated with Streptavidin-HRP for 30 min, washed again, and exposed to Chemi Reagent Mix for 1 min. Signals were visualized by chemiluminescence and quantified using FIJI software.

### Immunofluorescence staining

NK-92MI cells (5 × 10^5^) were seeded onto poly-L-lysine-coated coverslips and incubated at 37 °C in a 5% CO_2_ incubator for 30 min to allow attachment. Cells were then fixed with 4% paraformaldehyde for 15∼20 min at room temperature, permeabilized with 0.1% Triton X-100 for 5 min, and blocked with 1% bovine serum albumin (BSA) for 1 h at room temperature. For primary antibody incubation, samples were incubated overnight at 4 °C with antibodies against either total CDC42 (1:500; Santa Cruz Biotechnology, Cat. #sc-8401) or active CDC42 (1:500; NewEast Biosciences, Cat. #26905). After washes with PBS, cells were incubated with CoraLite^®^488-conjugated AffiniPure Goat Anti-Mouse IgG (H+L) secondary antibody (1:500; PTGLAB, Cat. #SA00013-1) for 1 h at room temperature in the dark. F-actin was stained with rhodamine-labeled phalloidin (1:200; Solarbio, Cat. #CA1610) for 30 min, and nuclei were counterstained with ready-to-use DAPI solution for 10 min, both performed in the dark with PBS washes between steps. Finally, coverslips were mounted cell-side down onto glass slides using an anti-fade mounting medium, sealed with nail polish, and stored at 4 °C in the dark until imaging. Images were acquired using a laser scanning confocal microscope, and the mean fluorescence intensity per cell was quantified using FIJI software.

### F-actin radial distribution analysis

For spatial analysis of F-actin distribution, fluorescence images of phalloidin-stained NK cells were analyzed using FIJI. Individual cells with clearly distinguishable boundaries were manually outlined on the merged fluorescence images to generate single-cell regions of interest (ROIs). Each ROI was then computationally divided into 10 normalized radial bins extending from the cell center to the cell edge. The normalized radial position was defined from 0 to 1, where 0 represented the cell center and 1 represented the outer cell boundary. Accordingly, the 10 radial bins corresponded to relative radial positions of 0–0.1, 0.1–0.2, 0.2–0.3, 0.3–0.4, 0.4–0.5, 0.5–0.6, 0.6–0.7, 0.7–0.8, 0.8–0.9, and 0.9–1.0.

For each radial bin, F-actin fluorescence intensity was quantified, including the mean fluorescence intensity and integrated fluorescence intensity. To characterize the radial distribution of F-actin independently of differences in total cellular fluorescence, the integrated intensity within each bin was expressed as a percentage of the total F-actin intensity of the corresponding cell. These normalized values were used to generate the radial distribution curves and heatmaps.

Three complementary parameters were further calculated for each individual cell to quantify the spatial enrichment of F-actin. The intensity-weighted radial position was calculated as Σ(I_i × r_i)/ΣI_i, where I_i represents the F-actin intensity in radial bin i and r_i represents the normalized radial position of that bin. Values closer to 1 indicate a more peripheral distribution of F-actin, whereas lower values indicate redistribution toward the cell center. The peripheral/central intensity ratio was calculated by dividing the mean F-actin intensity in the peripheral region (normalized radial position 0.5–1.0) by that in the central region (0–0.5). The edge/inner-cell intensity ratio was calculated by dividing the mean F-actin intensity in the outermost region (0.8–1.0) by that in the inner-cell region (0–0.8). Higher values of these two ratios indicate greater peripheral or cortical enrichment of F-actin, respectively. Each cell was analyzed individually and treated as one data point for subsequent statistical analysis.

### Protein depletion of ovarian cancer ascites

To evaluate the contribution of proteinaceous components, ovarian cancer ascites was subjected to centrifugal ultrafiltration using a 3-kDa molecular-weight-cutoff filter. The protein-depleted fraction was collected and used to treat NK cells under the same conditions as intact ascites. Protein concentration was measured before and after depletion to confirm efficient protein removal.

### Live-cell imaging of NK cell killing dynamics

NK cells and SKOV3 target cells were co-cultured at an effector-to-target (E) ratio of 10:1 and continuously monitored by live-cell imaging for 8 h. NK-cell interactions with individual SKOV3 target cells were tracked throughout the imaging period, and only NK cells that formed a discernible contact with a target cell were included in the subsequent analysis. Based on the interaction dynamics and the fate of the contacted target cell, NK-target interactions were classified into two functional outcomes: successful killing and sustained but non-cytolytic contact. Successful killing was defined as sustained NK-target cell contact followed by morphological contraction of the target cell and subsequent loss of membrane integrity, as indicated by propidium iodide (PI) influx. Sustained but non-cytolytic contact was defined as prolonged NK-target cell interaction without detectable target-cell death during the observation period.

For successful killing events, the time interval from immune synapse formation to target-cell death (Δt) was quantified. Immune synapse formation was defined as the establishment of stable contact between an NK cell and a SKOV3 target cell, whereas target-cell death was determined by loss of membrane integrity accompanied by PI influx. The percentages of successful-killing and sustained but non-cytolytic contact NK cells were calculated for each independent experiment, and Δt was quantified at the single-cell interaction level.

### Measurement of extracellular perforin and granzyme B by ELISA

NK cells were pretreated with ovarian cancer ascites and subsequently co-cultured with SKOV3 cells for 5 h. After co-culture, the culture supernatants were collected and centrifuged at 1,000 × g for 15 min to remove cells and cellular debris. Extracellular perforin and granzyme B (GZMB) levels were measured using a Human Perforin 1 (PF1) ELISA Kit (MLBIO, Shanghai, China; Cat. #ml105197) and a Human Granzyme B ELISA Kit (MLBIO, Shanghai, China; Cat. #ml060098), respectively, according to the manufacturer’s instructions. Briefly, 100 μL of standards or clarified culture supernatants was added to the antibody-precoated microplate wells and incubated at 37 °C for 1.5 h in the dark. The wells were then washed three times with 300 μL of 1× wash buffer, followed by incubation with 100 μL of biotinylated detection antibody for 1 h at 37 °C in the dark. After four washes, 100 μL of streptavidin-HRP working solution was added to each well and incubated for 30 min at 37 °C in the dark. The wells were washed four times, and the color reaction was developed by sequentially adding 50 μL of chromogenic solution A and 50 μL of chromogenic solution B, followed by incubation for 15 min at 37 °C in the dark. The reaction was terminated by adding 50 μL of stop solution, and absorbance was measured at 450 nm within 5 min using a microplate reader. Perforin and GZMB concentrations were calculated from their respective standard curves using a four-parameter logistic regression model.

### Transcriptomic analysis

Differential expression analysis was performed using the RNA-sequencing dataset from our previous study (7). Raw counts were analyzed with the DESeq2 package. P-values were adjusted using the Benjamini-Hochberg procedure to control the false discovery rate (FDR), reported as adjusted p-values (referred to as q-values in the text). Genes with |log_2_ fold change| ≥ 1 and q < 0.01 were considered significantly differentially expressed (DEGs). For visualization, we selected six GO biological process (BP) terms related to cytoskeletal regulation: regulation of actin cytoskeleton organization (GO:0032956), positive regulation of cytoskeleton organization (GO:0051495), microtubule cytoskeleton organization involved in mitosis (GO:1902850), regulation of microtubule cytoskeleton organization (GO:0070507), negative regulation of cytoskeleton organization (GO:0051494), and regulation of actin cytoskeleton reorganization (GO:2000249).

### Data normalization and correlation analysis

To assess the correlation between NK cell cytotoxicity and traction force under different experimental conditions, linear regression and Spearman’s rank correlation analyses were performed. To minimize batch effects and enable cross-condition comparisons, all values were normalized within each experimental set by dividing by the mean of the corresponding control group, with the control group normalized to 1. The strength of the linear relationship between variables was quantified using the coefficient of determination (*R*^2^) from linear regression, while monotonic associations were evaluated using Spearman’s correlation coefficient (*r*).

### Statistical analysis

Statistical analysis and graphical representation were performed using GraphPad Prism 9.5 (GraphPad Software, USA). Data are presented as mean ± standard error of the mean (SEM) from at least three independent experiments. Comparisons between two groups were made using unpaired Student’s *t*-tests. For comparisons involving multiple groups, one-way or two-way analysis of variance (ANOVA) was used as appropriate. ns, *, **, *** and **** refer to no significant difference, *p* < 0.05, *p* < 0.01, *p* < 0.001 and *p* < 0.0001, respectively.

## Data Availability

The names of the repository/repositories and accession number(s) can be found in the article/**Supplementary Material**. Additional data are available from the corresponding authors upon reasonable request.

## Supplementary material

Supplementary Figure 1 Granzyme B expression, CD107a expression, and extracellular cytotoxic effector molecule levels in NK cells are unaltered by ovarian cancer ascites pretreatment. **(A)** Flow cytometry gating strategy for analyzing the apoptosis ratio of SKOV3 cells (Q2+Q3) (1), the percentages of CD45^+^Perforin^+^ NK cells (2), and the percentages of CD45^+^GZMB^+^ NK cells (3). **(B, C)** Statistical results of percentages of GZMB^+^ NK cells **(B)** and mean fluorescence intensities (MFI) of GZMB in CD45^+^GZMB^+^ NK cells **(C)** after co-culture with SKOV3 cells for 5 hours under different E:T ratios (*n* = 3 independent experiments). **(D)** Statistical results of percentages of CD107a^+^ NK cells after co-culture with SKOV3 cells for 5 hours under different E:T ratios (*n* = 4 independent experiments). **(E, F)** Statistical results of extracellular perforin **(E)** and granzyme B **(F)** concentrations in NK and AS-NK cell culture supernatants (*n* = 7 independent experiments). ns refers to no significant difference in Two-way ANOVA analysis **(B–D)** or unpaired *t*-tests **(E, F)**. Data are presented as mean ± s.e.m.

Supplementary Figure 2 Quantification of NK cell migration in transwell assays. Data are presented as mean ± s.e.m. * refers to *p* < 0.05 in unpaired *t*-tests.

Supplementary Figure 3 Representative live-cell imaging sequence of an NK cell killing a SKOV3 cell. **(A)** Initial state with an NK cell and a SKOV3 cell. **(B)** Specific recognition and immune synapse formation between the cells. **(C)** Apoptotic membrane blebbing and contraction of the target cell. **(D)** Loss of membrane integrity indicated by propidium iodide (PI, red) influx, indicating cell death.

Supplementary Figure 4 Ascites treatment reduces CDC42 expression in NK cells. **(A–C)** Representative immunofluorescence images **(A)** and statistical results of F-actin **(B)** and CDC42 **(C)** expression in NK and AS-NK (scale bars = 5 μm; NK, *n* = 123; AS-NK, *n* = 102). Data are presented as mean ± s.e.m. **** refers to *p* < 0.0001 in unpaired *t*-tests.

Supplementary Figure 5 Representative flow cytometry plots of SKOV3 cell apoptosis after 5 h co-culture with NK-92MI cells under indicated conditions. Apoptosis was detected by Annexin V-FITC/PI staining. **(A)** NK vs. CN02-NK. **(B)** NK, AS-NK, and AS+CN02-NK. **(C)** NK vs. ZCL-NK. Q1: PI^+^ (dead); Q2: Annexin V^+^PI^+^ (late apoptotic/dead); Q3: Annexin V^+^PI^-^ (early apoptotic); Q4: viable. Apoptotic cells = Q2+Q3.

Supplementary Figure 6 Representative traction force microscopy heatmaps of NK-92MI cell traction stress under indicated conditions. **(A)** NK, AS-NK, and AS+CN02-NK cells. **(B)** NK and ZCL-NK cells. Color bars indicate traction stress in pascals (Pa).

Supplementary Figure 7 Effects of CDC42 pharmacological modulation on NK cell killing dynamics. **(A–F)** Comparisons of the percentage of successful-killing NK cells **(A, D)**, the percentage of sustained but non-cytolytic contact NK cells **(B, E)**, and the time from synapse formation to target-cell death **(C, F)** between DMSO and ZCL-NK groups **(A–C)** or between PBS and CN02-NK groups **(D–F)**. (**(A, B, D, E)** *n* = 3 independent experiments; **(C)** DMSO, *n* = 70 cells; ZCL-NK, *n* = 61 cells; **(F)** PBS, *n* = 47 cells; CN02-NK, *n* = 74 cells). Data are presented as mean ± s.e.m. ns, * and ** refer to no significant difference, *p* < 0.05 and *p* < 0.01, respectively, in unpaired *t*-tests.

Supplementary Figure 8 ZCL278-mediated CDC42 inhibition partially affects NK-cell effector functions. **(A–D)** Quantification of the percentages of perforin-positive NK cells **(A)**, IFN-γ concentrations in culture supernatants **(B)**, the percentages of GZMB-positive NK cells **(C)** and the percentages of CD107a-positive NK cells **(D)** before and after ZCL278 treatment. For **(A–C)**, *n* = 3. For **(D)**, *n* = 4. Data are presented as mean ± s.e.m. *ns* and * refer to no significant difference and *p* < 0.05, respectively, in unpaired *t*-tests.

Supplementary Figure 9 Effects of CDC42 pharmacological modulation on membrane protrusion formation and F-actin expression in NK cells. **(A)** Representative SEM images of DMSO, ZCL-NK, PBS, and CN02-NK cells. **(B–D)** Statistical analysis of mean F-actin intensity **(B)**, the mean length **(C)** and number **(D)** of membrane protrusions in DMSO vs. ZCL-NK cells (**(B)** DMSO, *n* = 37 cells; ZCL-NK, *n* = 30 cells; **(C, D)** *n* = 5 independent SEM images per group). **(E–G)** Statistical analysis of mean F-actin intensity **(E)**, the mean length **(F)** and number **(G)** of membrane protrusions in PBS vs. CN02-NK cells (**(E)** PBS, *n* = 68 cells; CN02-NK, *n* = 70 cells; **(F, G)** *n* = 5 independent SEM images per group). Data are presented as mean ± s.e.m. ns, *** and **** refer to no significant difference, *p* < 0.001 and *p* < 0.0001, respectively, in unpaired *t*-tests.

Supplementary Figure 10 Soluble component detection and KEGG pathway maps of NK cell function-related key factors. **(A)** Solid-phase antibody array detection of three independent ovarian cancer ascites samples. Each spot corresponded to a specific cytokine, chemokine, or soluble factor. **(B–D)**: Representative KEGG pathway maps for chemokine signaling **(B)**, TIM-3 **(C)**, and ICAM-1 **(D)**. Key molecules and associated processes are magnified in red.
