# Supplementary material for: Mechanical dysfunction: ovarian cancer ascites disables NK cells by suppressing CDC42-driven force generation
Source: Front Immunol. 2026 Oct 16;17:1883517. doi: 10.3389/fimmu.2026.1883517 (PMC13719564; doi:10.3389/fimmu.2026.1883517)
Supplement: Supplementary file 1 [file DataSheet1.docx]

**Supplementary Figures**

**Supplementary Figure 1. Granzyme B expression, CD107a expression, and extracellular cytotoxic effector molecule levels in NK cells are unaltered by ovarian cancer ascites pretreatment.** **A:** Flow cytometry gating strategy for analyzing the apoptosis ratio of SKOV3 cells (Q2+Q3) (1), the percentages of CD45⁺Perforin⁺ NK cells (2), and the percentages of CD45⁺GZMB⁺ NK cells (3). **B and C:** Statistical results of percentages of GZMB⁺ NK cells (B) and mean fluorescence intensities (MFI) of GZMB in CD45⁺GZMB⁺ NK cells (C) after co-culture with SKOV3 cells for 5 hours under different E:T ratios (*n* = 3 independent experiments). **D:** Statistical results of percentages of CD107a⁺ NK cells after co-culture with SKOV3 cells for 5 hours under different E:T ratios (*n* = 4 independent experiments). **E and F:** Statistical results of extracellular perforin (E) and granzyme B (F) concentrations in NK and AS-NK cell culture supernatants (*n* = 7 independent experiments). ns refers to no significant difference in Two-way ANOVA analysis (B–D) or unpaired *t*-tests (E and F). Data are presented as mean ± s.e.m.

**Supplementary Figure 2. Quantification of NK cell migration in transwell assays.** Data are presented as mean ± s.e.m. * refers to *p* < 0.05 in unpaired *t*-tests.

**Supplementary Figure 3. Representative live-cell imaging sequence of an NK cell killing a SKOV3 cell.** **A:** Initial state with an NK cell and a SKOV3 cell. **B:** Specific recognition and immune synapse formation between the cells. **C:** Apoptotic membrane blebbing and contraction of the target cell. **D:** Loss of membrane integrity indicated by propidium iodide (PI, red) influx, indicating cell death.

**Supplementary Figure 4. Ascites treatment reduces CDC42 expression in NK cells. A-C:** Representative immunofluorescence images (A) and statistical results of F-actin (B) and CDC42 (C) expression in NK and AS-NK (scale bars = 5 μm; NK, *n* = 123; AS-NK, *n* = 102). Data are presented as mean ± s.e.m. **** refers to *p* < 0.0001 in unpaired *t*-tests.

**Supplementary Figure 5. Representative flow cytometry plots of SKOV3 cell apoptosis after 5 h co-culture with NK-92MI cells under indicated conditions.** Apoptosis was detected by Annexin V‑FITC/PI staining. **A:** NK vs. CN02-NK. **B:** NK, AS-NK, and AS+CN02-NK. **C:** NK vs. ZCL-NK. Q1: PI⁺ (dead); Q2: Annexin V⁺PI⁺ (late apoptotic/dead); Q3: Annexin V⁺PI⁻ (early apoptotic); Q4: viable. Apoptotic cells = Q2+Q3.

**Supplementary Figure 6. Representative traction force microscopy heatmaps of NK-92MI cell traction stress under indicated conditions. A:** NK, AS-NK, and AS+CN02-NK cells. **B:** NK and ZCL-NK cells. Color bars indicate traction stress in pascals (Pa).

**Supplementary Figure 7. Effects of CDC42 pharmacological modulation on NK cell killing dynamics.** **A–F:** Comparisons of the percentage of successful-killing NK cells (A, D), the percentage of sustained but non-cytolytic contact NK cells (B, E), and the time from synapse formation to target-cell death (C, F) between DMSO and ZCL-NK groups (A-C) or between PBS and CN02-NK groups (D-F). (A, B, D, E: *n* = 3 independent experiments; C: DMSO, *n* = 70 cells; ZCL-NK, *n* = 61 cells; F: PBS, *n* = 47 cells; CN02-NK, *n* = 74 cells). Data are presented as mean ± s.e.m. ns, * and ** refer to no significant difference, *p* < 0.05 and *p* < 0.01, respectively, in unpaired *t*-tests.

**Supplementary Figure 8. ZCL278-mediated CDC42 inhibition partially affects NK-cell effector functions. A–D:** Quantification of the percentages of perforin-positive NK cells (A), IFN-γ concentrations in culture supernatants (B), the percentages of GZMB-positive NK cells (C) and the percentages of CD107a-positive NK cells (D) before and after ZCL278 treatment. For **A-C**, *n* = 3. For **D**, *n* = 4. Data are presented as mean ± s.e.m. *ns* and * refer to no significant difference and *p* < 0.05, respectively, in unpaired *t*-tests.

**Supplementary Figure 9. Effects of CDC42 pharmacological modulation on membrane protrusion formation and F-actin expression in NK cells. A:** Representative SEM images of DMSO, ZCL-NK, PBS, and CN02-NK cells. **B-D:** Statistical analysis of mean F-actin intensity (B), the mean length (C) and number (D) of membrane protrusions in DMSO vs. ZCL-NK cells (B: DMSO, *n* = 37 cells; ZCL-NK, *n* = 30 cells; C, D: *n* = 5 independent SEM images per group). **E-G:** Statistical analysis of mean F-actin intensity (E), the mean length (F) and number (G) of membrane protrusions in PBS vs. CN02-NK cells (E: PBS, *n* = 68 cells; CN02-NK, *n* = 70 cells; F, G: *n* = 5 independent SEM images per group). Data are presented as mean ± s.e.m. ns, *** and **** refer to no significant difference, *p* < 0.001 and *p* < 0.0001, respectively, in unpaired *t*-tests.

**Supplementary Figure 10. Soluble component detection and KEGG pathway maps of NK cell function-related key factors.** **A:** Solid-phase antibody array detection of three independent ovarian cancer ascites samples. Each spot corresponded to a specific cytokine, chemokine, or soluble factor. **B-D:** Representative KEGG pathway maps for chemokine signaling (B), TIM-3 (C), and ICAM-1 (D). Key molecules and associated processes are magnified in red.
